# Navigating technical hurdles: implementing a tailored Vac-Lok prone pillow technique to optimize radiotherapy in a case of large pendulous breast

**DOI:** 10.1093/bjrcr/uaaf059

**Published:** 2025-12-03

**Authors:** Pranav Nair, Ajay Sasidharan, Sruthi Kalavagunta, Prasath Bhaskaran, Alma Peter, Annex Edapattu Haridas, Debnarayan Dutta

**Affiliations:** Radiation Oncology, Amrita Institute of Medical Sciences, Kochi, India; Radiation Oncology, Amrita Institute of Medical Sciences, Kochi, India; Radiation Oncology, Amrita Institute of Medical Sciences, Kochi, India; Radiation Oncology, Amrita Institute of Medical Sciences, Kochi, India; Medical Physics, Amrita Institute of Medical Sciences, Kochi, India; Medical Physics, Amrita Institute of Medical Sciences, Kochi, India; Radiation Oncology, Amrita Institute of Medical Sciences, Kochi, India

**Keywords:** Vac-Lok, prone, breast, radiotherapy

## Abstract

Breast radiotherapy, typically administered using tangential fields in the supine position, employs various strategies to improve dose uniformity and minimize radiation exposure to sensitive organs. However, managing patients with large or pendulous breasts presents technical challenges, leading to the investigation of prone position radiotherapy, which has shown benefits in reducing skin reactions and lung radiation dose. Herein, we present a technical note detailing the implementation of a customized Vac-Lok prone pillow setup for adjuvant radiotherapy in a patient with extremely large pendulous breasts. The use of polystyrene foam slabs of 7 cm each and a Vac-Lok over it with a groove to place the ipsilateral breast hanging down and away from the midline and arms extended overhead on a customized prone pillow, enabled a comfortable position for the patient. Compared to the plan generated in the supine position, the prone plan showed a significant reduction in radiation dose to the ipsilateral lung.

## Introduction

Breast radiotherapy is typically administered utilizing tangential fields in the supine position. Various techniques, such as the application of higher energy photons, field-in-field intensity modulation, cardiac shielding, and breath-hold manoeuvres, are commonly employed to enhance dose homogeneity and minimize radiation exposure to organs at risk. Despite these efforts, individuals with large or pendulous breasts may still pose a significant challenge in radiotherapy planning and experience significant acute skin reactions due to the juxtaposition of the lower breast and inframammary skin, and also increased lung dose, particularly in cases where the breasts extend laterally beyond the midaxillary line.

Strategies for managing large pendulous breasts (>1600 cc) in the supine position include the utilization of breast strapping techniques with tapes or the creation of a custom mould mask designed to elevate the breast, thus mitigating contact with the inframammary skin fold and reducing the extension beyond the midaxillary line. However, it is essential to note that in instances where the skin is predisposed to inflammatory conditions (autoimmune disorders such as scleroderma, Sjogren’s etc), the application of these supportive devices may prove counterproductive, potentially leading to an exacerbation. Therefore, a thoughtful and individualized approach is warranted to address the unique challenges posed by large or pendulous breasts during supine-positioned breast radiotherapy, considering both anatomical factors and the condition of the patient’s skin.

The utilization of a prone position during breast radiotherapy simulation offers distinct advantages by causing the breast to move away from the chest wall and other critical underlying organs, thereby diminishing radiation exposure to the lung and heart.^1–3^ This positioning results in a narrowing of breast contours, contributing to a more uniformly distributed dose throughout the breast tissue. Various commercially available devices are available for the prone setup in breast radiotherapy. Most are applied for breast-alone radiotherapy, with some also having provision for regional node radiation. The adoption of a prone position comes with the drawback of discomfort experienced by patients lying on a firm surface. This is particularly pertinent for individuals with large pendulous breasts, a common characteristic among patients with elevated body mass index. Many individuals within this demographic, often comprising middle-aged or elderly individuals, present with additional comorbidities and respiratory challenges. These factors can contribute to discomfort during the maintenance of the prone position over an extended setup duration.

Given the potential discomfort associated with prone positioning, it is not widely used as the standard setup for breast radiotherapy. But especially in patients with large pendulous breasts and additional health considerations, it becomes imperative for healthcare practitioners to find personalized and patient-centred approaches in determining the most suitable radiotherapy positioning, taking into account both clinical efficacy and individualized patient comfort.

## Case description

A 49-year-old female patient was diagnosed with stage IIB (pT2 N1), grade 3, invasive breast carcinoma. Immunohistochemistry further identified the case as Luminal B-type breast cancer. The patient underwent a comprehensive therapeutic regimen, which included a wide local excision coupled with a sentinel lymph node biopsy, followed by adjuvant radiation therapy and hormones. Radiation was planned for the whole breast and regional nodes, including the supraclavicular fossa and the axillary apex, to a dose of 40 Gy in 15 fractions, followed by a sequential tumour bed boost of 10 Gy in 5 fractions.

The patient had large pendulous breasts, with lower reaching up to the lumbar region. Notably, in the supine position, lateral sagging of the breasts beyond the midaxillary line was observed, and a significant inframammary fold extended to the right lumbar region of the abdomen. Considering this alternative simulation set-up, options were explored beyond the conventional supine position on the breast board.

## Technical note

**Figure 1.**
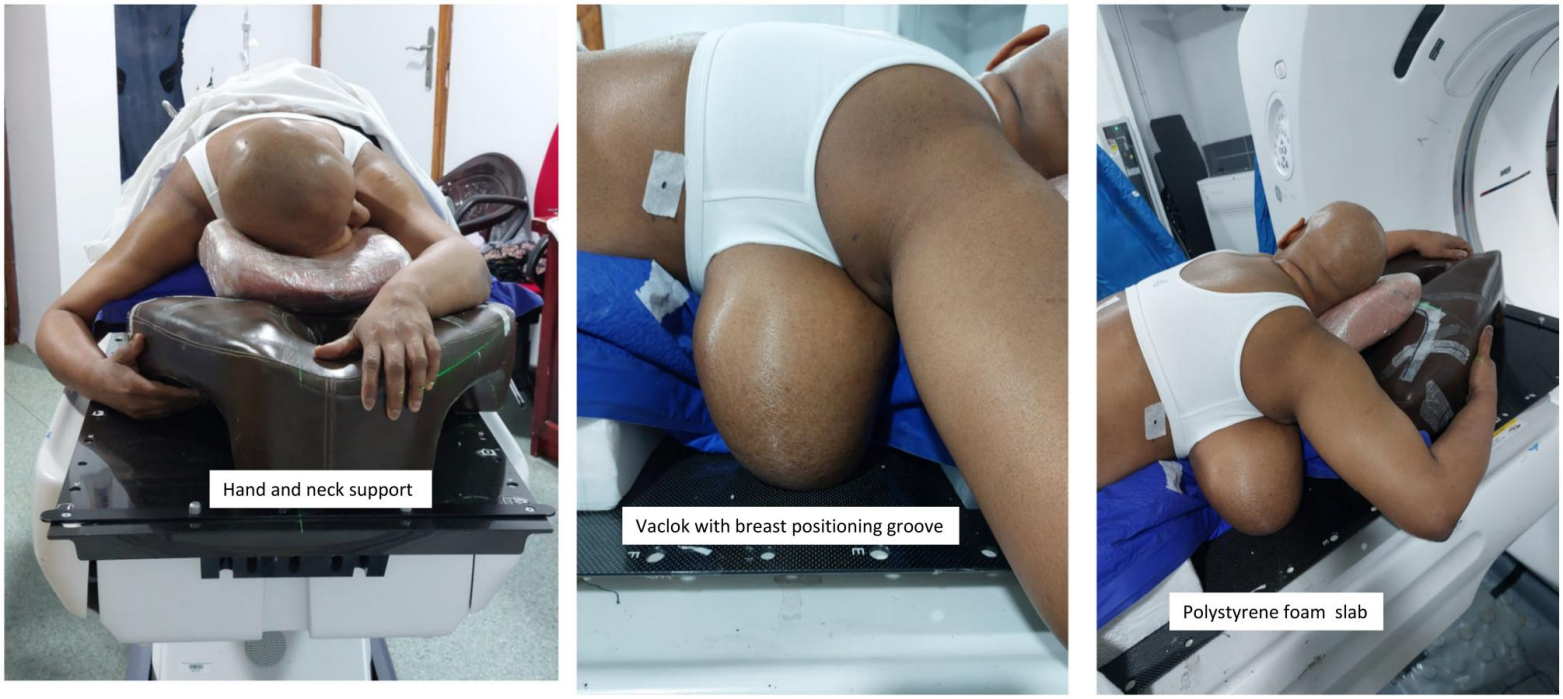
Patient positioned in the prone position using a customized Vac-Lok and a “prone pillow”.

For the prone setup, the patient assumed a position supported by a Vac-lok placed over 2 polystyrene foam slabs (with space for hanging the ipsilateral breast), each with a height of 7 cm. Both arms were extended above the head, and the hands were interlocked over an in-house customized prone pillow support to minimize body rotation. Notably, efforts were made to shift the contralateral breast away from the treated breast. Furthermore, the patient’s head was oriented away from the treated side. The ipsilateral breast hung down and laterally away from the midline, over the groove made on the vaclok (Figure 1).

The CT simulation was done using the Optima580WRT CT Simulator (GE Healthcare, Chicago, Illinois, USA). Images were acquired with a 2.5 mm slice thickness, covering the entire neck, thoracic, and upper abdomen regions—from the infraorbital margin to the L2 vertebra. During the CT simulation process, a posterior-anterior setup point was established, and 3 levelling markers were placed on the skin. These markers included one positioned on the midline in the dorsal region, another located at the level of the lower aspect of the scapula, and a third situated at the level of the mid-axillary line. CT simulation was also done in the supine position to create a plan for dosimetric comparison. The patient was positioned with both arms elevated above the head, utilizing a Med-TecM350 breast board (Civco Radiotherapy, Orange City, Iowa, USA) set at an inclination of 30°.

The contouring included the clinical target volume (CTV), planning target volumes (PTV), and ipsilateral lung. The CTV was defined to encompass the entire breast tissue, commencing 5 mm below the skin surface. The “PTV breast eval” was derived by incorporating a 10 mm margin to the CTV and subsequent adjustments from field margins and the skin. The planning was done through the Monaco Treatment Planning System Version 5.11. The treatment strategy involved the utilization of 2 opposed tangential fields employing 6 MV photon beams for the whole breast. Regional nodes were covered using postero-anterior oblique fields. Customization of radiation fields was implemented as necessary through multileaf collimators to mitigate exposure to adjacent healthy tissues. Beam angles were meticulously adjusted to minimize irradiation of the lung parenchyma. The field-in-field technique was employed to optimize target coverage and mitigate dose heterogeneity. The prescribed total dose amounted to 40 Gy administered over 15 fractions through the 2 tangential fields, followed by a boost dose of 10 Gy in 5 fractions directed to the tumour bed, delivered via mini tangents. The dose calculation process employs a grid resolution of 3 mm, with collapsed cone convolution algorithm. Subsequently, dose-volume histograms (DVHs) were generated for critical structures, namely the clinical target volume (CTV), planning target volumes (PTV), ipsilateral lung, heart, and the left anterior descending coronary artery. These DVHs provided a comprehensive visualization of the dose distribution within these structures, aiding in the assessment and optimization of the treatment plan concerning established dose constraints and clinical objectives.

**Figure 2.**
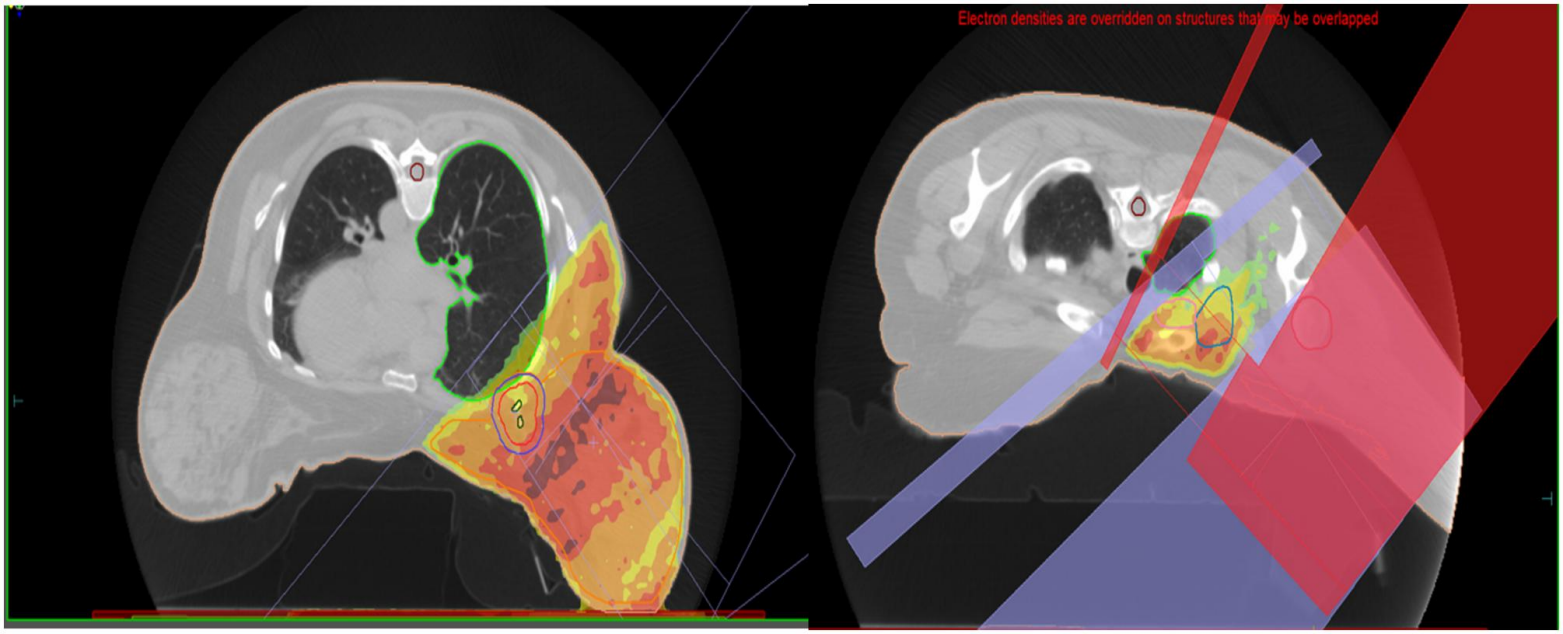
Whole breast and regional node radiotherapy plan in the prone position.

**Figure 3.**
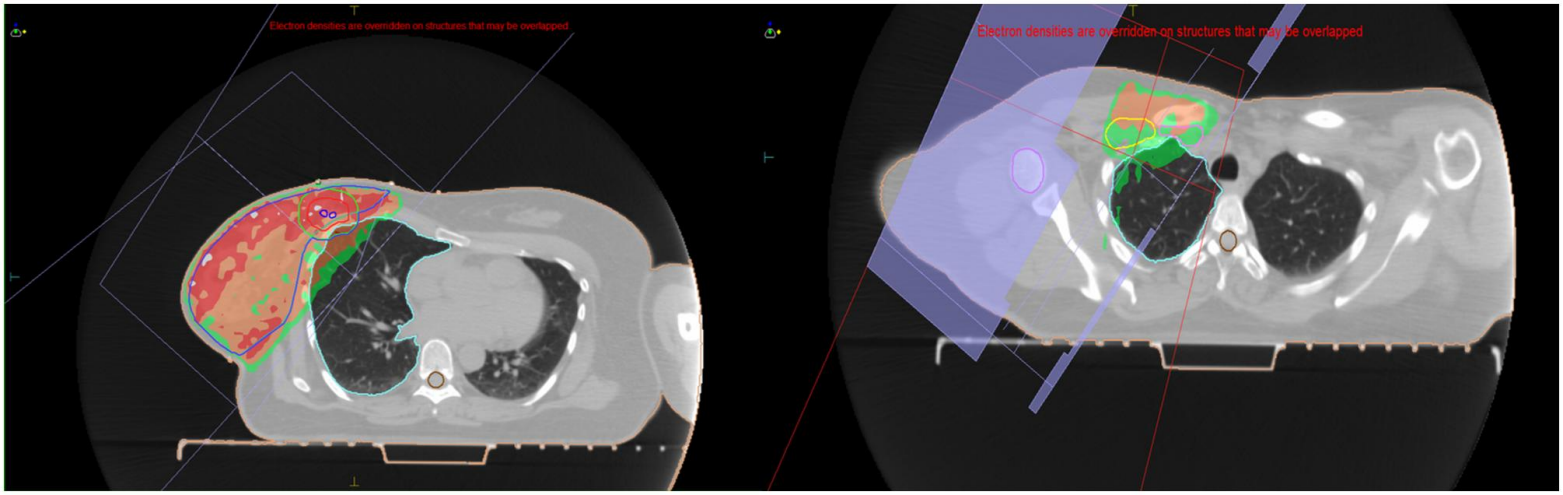
Whole breast and regional node radiotherapy plan in the supine position.

For each case, 2 distinct treatment plans were made, for the prone and supine positions, respectively (Figures 2 and 3). The prioritization in both scenarios was directed towards optimizing target coverage while adhering to established dose limitations for lung parenchyma, specifically maintaining the volume of lung receiving 17 Gy below 35%. The acceptable criteria for the PTV eval coverage were set at D_95_ greater than 90% and V_105_ less than 5%. The dosimetric parameters attained in the prone and supine positions are comprehensively detailed in Tables 1 and 2. Notably, a noteworthy reduction in lung dose was observed, with a mean dose of 8.2 Gy in the prone position to a dose of 13.9 Gy in the supine position. There was no significant difference in mean heart dose or maximum skin dose between the 2 positions.

**Table 1.** Dosimetric parameters for target volumes.

A) Prone position									
Structure	Volume (cm^3^)	Min. dose (cGy)	Max. dose (cGy)	Mean dose (cGy)	D90%	D95%	D2%	V105%	Heterogeneity Index
PTV-right breast	1869.41	2819.8	4475.6	3956.6	3773.9	3724.9	4227.3	3.79%	1.12
PTV-tumour bed	57.95	3525.1	4282.7	3839.9	3723.8	3697.7	4058.8	0.13%	1.09
Right level III nodes	10.841	3552.6	4270.6	3903.0	3770.7	3743.1	4142.9	0.3%	1.09
Right level II nodes	11.679	2292.2	4054.4	3786.2	3677.3	3577.5	3978.0	0%	1.10
Right level I nodes	47.215	3440.6	4043.3	3748.7	3661.9	3622.5	3885.6	0%	1.06
Right supraclavicular fossa	12.603	3013.0	4267.4	3912.1	3711.3	3629.1	4139.1	0.34%	1.13

B) Supine position									
Structure	Volume (cm^3^)	Min. dose (cGy)	Max. dose (cGy)	Mean dose (cGy)	D90%	D95%	D2%	V105%	Heterogeneity Index
PTV-right breast	1765.74	2676.6	4481.6	3925.0	3741.1	3671.1	4207	2.29	1.13
PTV-tumour bed	68.283	3526.5	4426.0	4032.0	3865.9	3822.8	4279.4	10.45	1.11
Right level III nodes	8.946	3586.4	4100.8	3868.7	3760.3	3725.0	4024.3	0	1.06
Right level II nodes	25.267	2337.1	4072.9	3792.3	3670.3	3616.4	3970.5	0	1.09
Right level I nodes	40.105	3314.8	4013.2	3691.2	3541.7	3473.7	3895.3	0	1.11
Right supraclavicular fossa	18.817	2980.1	4219.8	3814.2	3632.5	3527.9	4035.0	0.01	1.14

**Table 2.** Dosimetric parameters achieved for organs at risk.

A) Prone position					
Structure	Volume (cm^3^)	Mean dose (cGy)	Min. dose (cGy)	Max. dose (cGy)	Volume-based constraint
**Heart**	504.28	79.4	19.2	304.4	V2Gy = 2.36%
**Right lung**	1355.27	822.9	27.7	4028.5	V17Gy = 20.12%
**Spinal cord**	26.24	59.6	3	252.8	–
**Skin**	–	–	–	4385.5	V42Gy = 4.4 cc

B) Supine position					
Structure	Volume (cm^3^)	Mean dose (cGy)	Min. dose (cGy)	Max. dose (cGy)	Volume-based constraint
**Heart**	521.5	60.5	15	304.4	V2Gy = 0.15%
**Right lung**	1203.8	1346.3	0	4149.2	V17Gy = 34.15%
**Spinal cord**	33.83	73.1	20.8	386.6	–
**Skin**	–	–	–	4341.0	V42Gy = 0.79 cc

During the treatment session, portal images capturing the 2 tangential treatment fields were obtained and subsequently co-registered with the digitally reconstructed radiographs outlined in the treatment plan. Interfraction reproducibility was ensured during the entire treatment course, using daily positional setup verification and twice weekly portal imaging verification, which assured minimal variation during daily setups for treatment. To assess the acute toxicity, the Radiation Therapy Oncology Group criteria were employed, with evaluations conducted weekly throughout the treatment course. Notably, the patient exhibited only grade I skin toxicity after the radiation treatment, indicating a favourable and well-tolerated therapeutic outcome.

## Discussion

The contemporary practice uses supine positioning for adjuvant radiotherapy in breast cancer patients, yet the anatomical advantages inherent in the prone position, particularly in mitigating heart and lung toxicity, are acknowledged. Despite evidence supporting the safety, feasibility, and dosimetric advantages of the prone position, its utilization remains notably limited in modern radiotherapy practices.^4^ A German study underscored this discrepancy, revealing that only one out of 68 surveyed radiotherapy centres incorporated heart-sparing techniques through prone positioning.^5^ Similarly, findings from the Michigan Radiation Oncology Quality Consortium disclosed that merely 4.3% of 4688 breast cancer patients received prone treatment.^6^ In a dosimetric database review from Brisbane, Australia, the prone position was adopted in only 1.8% of cases, contrasting with 708 cases treated in the supine position.^7^

The reluctance to embrace the prone position is attributed to the intricacies involved in its implementation and the availability of commercially prone setup platforms. This setup necessitates the breast to hang down from the chest wall through an aperture on the support couch, enabling the use of tangential fields to spare organs in the thoracic cavity, such as the lung, heart, and oesophagus. Challenges encompass patient comfort, stability, and precision in setup using current devices, often leading to extended setup times, especially when incorporating regional node radiotherapy.^8^ Setup discomfort is reported in the literature as a common drawback for prone positioning in breast radiotherapy.^9–11^ However, in the presented case, this challenge is effectively addressed by employing a customized Vac-lok prone pillow setup, with the patient’s hands positioned above the head. Notably, the patient experienced comfort throughout the entire treatment course, and no treatment breaks were necessitated due to positioning issues. This setup procedure is a suitable alternative to flat-prone platforms, increasing comfort for the patient.

Additionally, information regarding the dosimetric advantages of the prone position in right-sided breast cancers remains limited. A study conducted in Geneva aimed to assess the dosimetric benefits of transitioning from the supine to the prone position in right whole breast radiotherapy.^12^ Advantage, in this context, was defined as achieving the lowest radiation dose to non-target organs while delivering the prescribed dose to the tumour bed and ipsilateral breast. The advantage score was quantified using the penalty score, previously analysed in left-sided breast cancers.^13^ The study identified 146 patients with dual plans for the right breast. In 81.5% of cases (119 patients), the penalty score was reduced when comparing the prone to supine positions, accompanied by a substantial 70.8% reduction in lung doses. The authors noted that among patient characteristics, breast volumes were the only significant predictors, and no specific cut-off could determine when supine positioning would be more advantageous than prone. This advantageous trend was evident in our patient, with a 38.9% reduction in the mean lung dose when transitioning from supine to prone (*D*_mean/supine_ = 13.46 Gy, *D*_mean/prone_ = 8.22 Gy). The study also highlighted that this benefit is independent of breast pendulousness, as in right breast radiotherapy, the distance between the tangential field’s edge and the heart is less influenced by how the gantry is adapted to the breast.

Information regarding the role of the prone position in regional nodal irradiation remains limited. A study conducted by Shin et al^14^ in New York, United States of America, included 69 patients with breast cancer. The study reported lower lung and heart doses in the prone position, even among patients who underwent nodal irradiation, as compared to the supine position. In our patient, who was treated in the prone position, there was satisfactory coverage of the three axillary nodal regions as well as the supraclavicular regions. A limitation of this setup is that the beam has to traverse through the couch and support materials. An alternative approach to this setup is the prone crawl position, as described by Deseyne et al from a centre in Belgium.^8^ The crawl breast couch permits beam access from underneath the patient without traversing support materials, allowing for adequate target coverage of all the regional nodes, except the intramammary nodes.

A study by Song et al^15^ analysed the setup reproducibility difference between the Vac-lok bag and thermoplastic mask for breast radiotherapy, with all patients in both setups being treated in the supine position. They observed that patients treated with the vacuum lock experienced increased comfort levels. Also, Kainz et al^16^ investigated the role of the prone position in helical tomotherapy for simultaneous breast and regional nodal irradiation. Their findings indicated that mean doses to the contralateral lung and heart were lower for right breast cases compared to left breast cases. Additionally, the mean organ doses to the ipsilateral lung and contralateral breast from the prone breast tomotherapy plans were similar to those reported for conventional techniques. The focal point of this report is the adoption of a customized prone position utilizing the Vac-Lok and prone pillow. To comprehensively assess the feasibility of using the Vac-Lock in the prone position, a larger cohort of patients would be required for further analysis.

## Conclusion

The Vaclok-prone pillow setup was used for optimal planning of breast and regional node radiotherapy in a large and pendulous breast. This report notes the technical feasibility, the advantage of reducing the lung dose, and the enhanced patient comfort associated with this approach.

## Consent

Written informed consent was obtained from the patient for publication of this case, including accompanying images.

## References

[1] Formenti SC , DeWyngaert JK, Jozsef G, Goldberg JD. Prone vs supine positioning for breast cancer radiotherapy. JAMA. 2012;308:861-863. 10.1001/2012.jama.10759 22948692

[2] Osa E-OO , DeWyngaert K, Roses D, et al Prone breast intensity modulated radiation therapy: 5-year results. Int J Radiat Oncol Biol Phys. 2014;89:899-906. 24867535 10.1016/j.ijrobp.2014.03.036 PMC4684090

[3] Varga Z , Cserháti A, Rárosi F, et al Individualized positioning for maximum heart protection during breast irradiation. Acta Oncol. 2014;53:58-64. 23544358 10.3109/0284186X.2013.781674

[4] Veldeman L , Schiettecatte K, De Sutter C, et al The 2-year cosmetic outcome of a randomized trial comparing prone and supine whole-breast irradiation in large-breasted women. Int J Radiat Oncol Biol Phys. 2016;95:1210-1217. 27209501 10.1016/j.ijrobp.2016.03.003

[5] Duma MN , Münch S, Oechsner M, Combs SE. Heart-sparing radiotherapy in patients with breast cancer: what are the techniques used in the clinical routine? A pattern of practice survey in the German-speaking countries. Med Dosim. 2017;42:197-202. 28502653 10.1016/j.meddos.2017.03.002

[6] Pierce LJ , Feng M, Griffith KA; Michigan Radiation Oncology Quality Consortium, et al Recent time trends and predictors of heart dose from breast radiation therapy in a large quality consortium of radiation oncology practices. Int J Radiat Oncol Biol Phys. 2017;99:1154-1161. 28927756 10.1016/j.ijrobp.2017.07.022

[7] Kairn T , Crowe SB. Application of retrospective data analysis to clinical protocol design: can the potential benefits of breath-hold techniques for breast radiotherapy be assessed without testing on patients? Australas Phys Eng Sci Med. 2019;42:227-233. 30848439 10.1007/s13246-019-00725-w

[8] Deseyne P , Speleers B, De Neve W, et al Crawl positioning improves set-up precision and patient comfort in prone whole breast irradiation. Sci Rep. 2020;10:16376. 33009448 10.1038/s41598-020-72702-3 PMC7532156

[9] Lai J , Zhong F, Deng J, et al Prone position versus supine position in postoperative radiotherapy for breast cancer: a meta-analysis. Medicine (Baltimore). 2021;100:e26000. 34011096 10.1097/MD.0000000000026000 PMC8136988

[10] Hannan R , Thompson RF, Chen Y, et al Hypofractionated whole-breast radiation therapy: does breast size matter? Int J Radiat Oncol Biol Phys. 2012;84:894-901. 22543209 10.1016/j.ijrobp.2012.01.093

[11] Vesprini D , Davidson M, Bosnic S, et al Effect of supine vs prone breast radiotherapy on acute toxic effects of the skin among women with large breast size: a randomized clinical trial. JAMA Oncol. 2022;8:994-1000. 35616948 10.1001/jamaoncol.2022.1479 PMC9136674

[12] Fargier-Bochaton O , Wang X, Dipasquale G, et al Prone versus supine free-breathing for right-sided whole breast radiotherapy. Sci Rep. 2022;12:525. 35017568 10.1038/s41598-021-04385-3 PMC8752750

[13] Wang X , Fargier-Bochaton O, Dipasquale G, et al Is prone free breathing better than supine deep inspiration breath-hold for left whole-breast radiotherapy? A dosimetric analysis. Strahlenther Onkol. 2021;197:317-331. 33416915 10.1007/s00066-020-01731-8 PMC7987627

[14] Shin SM , No HS, Vega RM, et al Breast, chest wall, and nodal irradiation with prone set-up: results of a hypofractionated trial with a median follow-up of 35 months. Pract Radiat Oncol. 2016;6:e81-8-e88. 26723552 10.1016/j.prro.2015.10.022

[15] Song Y , Peng J, Chen Q, Luo H. Compare of interfractional setup reproducibility between vacuum-lock bag and thermoplastic mask in radiotherapy for breast cancer. Technol Cancer Res Treat. 2021;20:15330338211043037. 34554027 10.1177/15330338211043037 PMC8490727

[16] Kainz K , White J, Chen GP, Hermand J, England M, Li XA. Simultaneous irradiation of the breast and regional lymph nodes in prone position using helical tomotherapy. Br J Radiol. 2012;85:e899-905-e905. 22457317 10.1259/bjr/18685881 PMC3474030

